# Comprehensive In Vitro Testing of Calcium Phosphate-Based Bioceramics with Orthopedic and Dentistry Applications

**DOI:** 10.3390/ma12223704

**Published:** 2019-11-10

**Authors:** Radu Albulescu, Adrian-Claudiu Popa, Ana-Maria Enciu, Lucian Albulescu, Maria Dudau, Ionela Daniela Popescu, Simona Mihai, Elena Codrici, Sevinci Pop, Andreea-Roxana Lupu, George E. Stan, Gina Manda, Cristiana Tanase

**Affiliations:** 1 Victor Babes National Institute of Pathology, Biochemistry-Proteomics Department, 050096 Bucharest, Romania; radu_a1@yahoo.com (R.A.); albulescu.l@gmail.com (L.A.); dudau_maria2002@yahoo.com (M.D.); danabini@yahoo.com (I.D.P.); simona.mihai21@gmail.com (S.M.); raducan.elena@gmail.com (E.C.); sevincipop@yahoo.com (S.P.); ldreea@gmail.com (A.-R.L.); gina.manda@gmail.com (G.M.); 2 Department Pharmaceutical Biotechnology, National Institute for Chemical-Pharmaceutical R&D, 031299, Bucharest, Romania; 3 National Institute of Materials Physics, 077125 Magurele, Romaniageorge_stan@infim.ro (G.E.S.); 4 Army Centre for Medical Research, 010195 Bucharest, Romania; 5 Department of Cellular and Molecular Biology and Histology, Carol Davila University of Medicine and Pharmacy, 050047 Bucharest, Romania; 6 Cantacuzino National Medico-Military Institute for Research and Development, 050096 Bucharest, Romania; 7 Cajal Institute, Titu Maiorescu University, 004051 Bucharest, Romania

**Keywords:** bioceramics, in vitro testing, hydroxyapatite, angiogenesis, osteogenesis, signaling pathways, microRNA

## Abstract

Recently, a large spectrum of biomaterials emerged, with emphasis on various pure, blended, or doped calcium phosphates (CaPs). Although basic cytocompatibility testing protocols are referred by International Organization for Standardization (ISO) 10993 (parts 1–22), rigorous in vitro testing using cutting-edge technologies should be carried out in order to fully understand the behavior of various biomaterials (whether in bulk or low-dimensional object form) and to better gauge their outcome when implanted. In this review, current molecular techniques are assessed for the in-depth characterization of angiogenic potential, osteogenic capability, and the modulation of oxidative stress and inflammation properties of CaPs and their cation- and/or anion-substituted derivatives. Using such techniques, mechanisms of action of these compounds can be deciphered, highlighting the signaling pathway activation, cross-talk, and modulation by microRNA expression, which in turn can safely pave the road toward a better filtering of the truly functional, application-ready innovative therapeutic bioceramic-based solutions.

## 1. Introduction

Due to their physical–chemical similarity with bone mineral, calcium phosphate (CaP) bioceramics, with prominent exponents hydroxyapatite (HA, Ca_10_(PO_4_)_6_(OH)_2_) and β-tricalcium phosphate (β-TCP, Ca_3_(PO_4_)_2_), are already used or envisaged to be employed, in pure or blended form, in a continuously increasing number of biomedical applications with the main focus on tissular regeneration within the skeletal system (bones, joints, and teeth) [1,2]. If used for orthopedic and dental transplants, CaPs may interact directly with the surrounding tissue, either supporting tissue growth or inducing tissue regeneration, while presenting a good compatibility with the biological systems [3,4,5]. Synthetic ceramics such as HA and β-TCP possess excellent biocompatibility and can interact directly with surrounding tissues through the formation of chemical and biochemical bonds [6,7]. Although many different type of materials were investigated and engineered (e.g., metallic, polymeric, or ceramic; natural or synthetic; bioinert, bioresorbable, or bioactive) to fabricate bone regeneration scaffolds or implant coatings, bioactive ceramics prominently arose as materials of choice due to their remarkable ability to create a strong bond with hard tissues, as well as prevent their encapsulation in fibrillary connective tissue, in contrast to renown bioceramics such as alumina or yttria, polymers, and metals [8,9,10,11]. Their bioactivity could also enhance gene activation for osteogenesis and angiogenesis. These materials evolved into an integral and vital segment of the modern healthcare system, and they can be integrated into the human body as permanent biomedical devices, due to their improved biocompatibility [12]. However, pure HA has limited usage in biomedicine because of its fragility and overall weak mechanical properties, unsuitable for developing load-bearing biomedical applications [13]. Nevertheless, the designed doping of CaP bioceramics with various cations (e.g., Na^+^, Mg^2+^, Sr^2+^, and Zn^2+^) or anions (e.g., (CO_3_)^2−^, (SiO_4_)^4−^, F^−^, and Cl^−^) have now the potential to transform them into major candidates for the future development of “smart materials”, due to their capability to combine biocompatibility and mechanical performance with specific effects such as antimicrobial activity, angiogenesis induction, and drug delivery capacity [8,9,10]. When designed as resorbable biomaterials with various resorption kinetics (spanning from days to months), their ion dissolution products (Ca^2+^, (PO_4_)^3−^, Na^+^, Si4^+^, Mg^2+^, and Sr^2+^ ions) can usually be processed via normal metabolism [14] or can even be exploited to exert desired therapeutic effects, such as the promotion of angiogenesis or osteogenesis properties, and antimicrobial activity [11,15]. This new generation of CaP-based materials is envisaged to be employed in healthcare in various shapes and forms: bulk (especially for bone graft substitutes, e.g., porous scaffolds) [16,17,18], highly crystalline or nano-structured implant coatings [19], and dispersed nanoparticles (e.g., as antimicrobials or carriers in biological systems for drug delivery, transfection, gene silencing, or imaging) [20,21,22] or nano-objects (e.g., nano-rods, nano-wires, nano-tubes, and nano-needles) [23,24,25,26].

Capitalizing on their osteoconductivity and biocompatibility [27], recent studies reported that nano-sized pure and cation- and/or anion-substituted HAs could represent promising candidates for bone regeneration, as they closely mimic the structural and compositional features of the inorganic component of native bone matrix [1,28,29]. Bone tissue engineering emerged as a rapidly developing strategy for bone regeneration due to the increased clinical demand for biocompatible bone scaffolds and novel biomaterials, in orthopedic and dental medicine [30,31,32].

Bone healing requires a plethora of biological intricately linked events, such as angiogenesis, osteogenesis, and inflammatory reactions, in order to stimulate the complex regeneration processes [33]. A comprehensive in vitro testing of newly developed bioactive materials should inquire at least these properties. The advanced testing should also assess gene modulation or gene toxicity since, in some cases, implants based on bioactive ceramics should function properly for long periods of time (sometimes exceeding one decade).

Currently, the biomaterials scientific community is quite hyperactive in this respect, producing an immense quantity of information, especially in the realm of cation- and/or anion-substituted CaPs. Although extensively characterized from compositional, morphological, and structural points of view, the pure and substituted CaPs were evaluated to a lesser extent from a biological point of view. Regularly, such studies only tackled the topic superficially or incompletely, limited to the evaluation of the biomineralization capacity [34,35,36] (https://www.iso.org/standard/65054.html) and cytotoxicity/cytocompatibility assays which do not provide in-depth understanding of biological processes and hinder, therefore, a rapid transition from bench to bedside. Unfortunately, only few such materials reach an advanced in vitro biological assessment stage, and even fewer get to the stage of in vivo testing as a prerequisite for biomaterial transfer in medicine [1]. 

The scope of this article is to review the actual state of the in vitro safety assessment methods, applicable for bioceramics (with a focus on CaP-based compounds), stressing the critical aspects of commonly used procedures/regulations and recommending the ways to improve the selection algorithm of such biomaterials for the best biological outcome when implanted in vivo. Although progress was recently recorded in the accuracy, complexity, and fastness of biological testing of bioceramics, no complete review on the achieved progress was published to the best of our knowledge. Such a breviary study could popularize the forefront technologies (which become increasingly available)/biological protocols, as well as their judicious coupling, and reorient the focus of the biomaterials community toward the insightful and comprehensive understanding of the biological mechanisms of their materials, rather than toward a simple screening of seldom functionalities. The application of in vitro methods to evaluate the eventual deleterious effects of materials (such as cytotoxicity, genotoxicity, or production of reactive oxygen species) is also highlighted. Certain attention was dedicated to the biofunctional analysis of bioceramics, offering information on the state-of-the-art methods for the evaluation of two key factors: angiogenesis and osteogenesis. Moreover, it is suggested that deciphering the mechanisms of action of these bioceramic CaP compounds can be accomplished by involving the specific modulation of microRNAs (miRNAs) and cell signaling within osteogenesis and dentinogenesis.

## 2. Biocompatibility Assessment of CaP-Based Bioceramics

### 2.1. Regulatory Aspects

**Figure 1:**
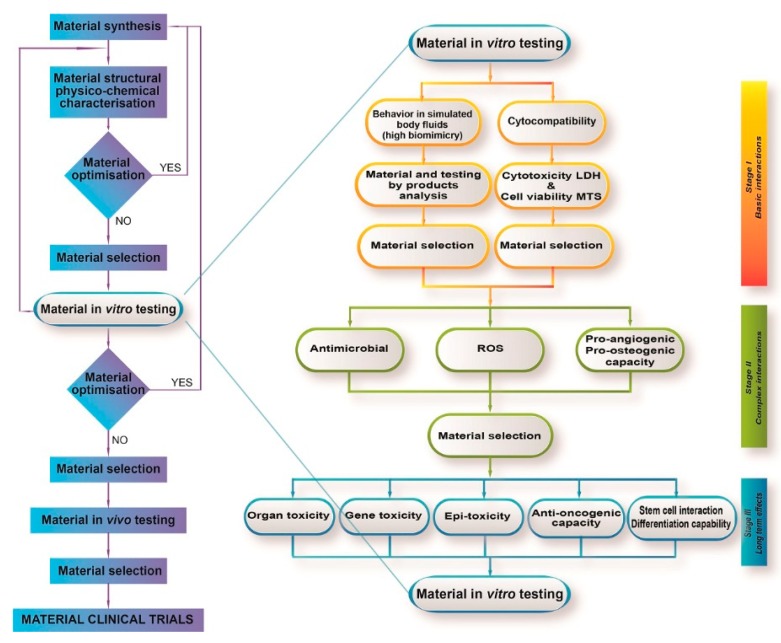
Workflow for selection of calcium phosphate (CaP)-based bioceramics suitable for patient applications. The left diagram is a general workflow diagram, from material synthesis to clinical application. In vitro testing is detailed in the right panel.

There is a complex set of International Organization for Standardization (ISO) standards governing the evaluation of biocompatibility. According to ISO 10993-1:2018 “Evaluation and Testing within a Risk Management Process”, a set of mandatory tests must be selected, depending on the nature and way of contact of the biomaterial/nanomaterial with the body [37]. In connection with ISO 10993-1:2018, a set of assays should be considered when checking for the biocompatibility of biomaterials, for both types of materials of interest, i.e., external communicating devices coming in contact with tissue, bone or dentin and internal communicating ones of similar uses. The ISO:10993-1:2018 recommends running in vitro assays regarding cytotoxicity, genotoxicity, immunotoxicity, and material-mediated pyrogenicity, as well as a series of in vivo assays regarding irritation, subacute/sub-chronic toxicity, and implantation. A general workflow for material testing, also applicable to CaP bioceramics, with emphasis on in vitro testing, is presented in Figure 1.

While these recommendations regard all kind of materials (in bulk or in thin-film form), some specific provisions are made for nano-powders and nano-objects (e.g., (i) particulate bioceramics of various designed nano-shapes, or (ii) nano-debris as a result of in situ wear/degradation of the biomedical device). Such nano-debris can be generated during the life cycle of a medical device; therefore, the evaluation of possible adverse effects caused by the implantation or generation of nano-objects, whether during preparation or in situ use, wear, or degradation of medical devices, needs to be addressed. This applies to medical devices having the potential to generate nanoscale wear and/or degradation particles. For the biological evaluation of medical devices, knowledge on the potential generation and/or release of nano-objects from such materials and on their effects is essential [3,8,38]. Such an example of release of nanoparticles (NPs) is provided, for instance, for titanium implants, discussed by Kim et al. [39], who reviewed the literature and found that particles and ions from titanium alloys can deposit in surrounding tissue, mainly because of the corrosion and wear of implants, further causing bone loss, and failure of osteointegration.

The procedures for the biological evaluation of medical devices, described in the ISO 10993 series of standards, can also be applied for the biological evaluation of medical devices containing nano-objects (e.g., nano-powders, nano-rods, nano-wires, nano-tubes, and nano-needles) as an integrated part of the device (e.g., HA nanowire/collagen [24], or HA nano-needle/poly(l-lactic acid [26] composite scaffolds, and CaP nanoparticles with an intrinsic antimicrobial effect [20]). However, when the release of nano-objects from the medical device is possible, a safety evaluation should also be performed on these released nanoscale entities. Furthermore, in addition to evaluating a medical device as a whole, its nanoscale components or constituents should also be separately assessed.

With regard to in vitro cytotoxicity assays, which are fast and reliable filters for bioceramics with undesired effects, several approaches are available. The main advantage is that such assays are not limited to certain cell lines; thus, a large variety of non-human mammalian and human cells can be employed. Moreover, the testing is not limited to “immortal” or “immortalized” cells; in certain situations, it can also be achieved using primary cells. These last two variants (immortalized and primary cells) were recently enhanced by quite a wide offer of such products, mainly from the American Type Culture Collection (ATCC). ISO 10993-5:2009 “Tests for Cytotoxicity—In Vitro Methods” stands as a regulatory document for these assays [40]. 

The ISO standard provides a large set of assays that can be applied in order to assess the potential acute adverse (toxicological) effects of extractables from medical device materials on mammalian cells, using the settings outlined in ISO 10993-5. While there is no explicit limitation with regard to the mammalian cell line to be used, the most frequent models are based on mouse or human cells. The standard testing procedures involve cell monolayers, grown near confluence in adequate recipients, exposed to extracts or eventually to biomaterials per se, under standard conditions, for various times (usually 24–72 h). Different techniques are employed for examination, starting from visual inspection under microscope (which allows for the evaluation of change in size and number of cell organelles, or disruption), viability assessments, such as lactate dehydrogenase (LDH), 3-(4,5-dimethylthiazol-2-yl)-2,5-diphenyltetrazolium bromide (MTT), 3-(4,5-dimethylthiazol-2-yl)-5-(3-carboxymethoxyphenyl)-2-(4-sulfophenyl)-2*H*-tetrazolium (MTS), etc., quantitation, and estimation of cell apoptosis or necrosis using specific assay methods. Usually, ISO 10993-5:2009 advises the use of cells from recognized repositories. Various culture types can be used, such as primary cell cultures, cell lines, or organotypic cultures, provided that accuracy and reproducibility of the response can be demonstrated [40]. 

There were several studies that demonstrated that HA and its related dissolution products could be quite harmless for use in devices, such as, for instance, bone implants [41,42,43]. However, some other studies still considered the potential risks of such materials, mainly due to the addition of other “doping” elements, such as Sr, Zn, etc. While in low concentrations they seem to be harmless, the increase of their content can result in strong modifications of the crystal lattice, leading to undesired effects (e.g., rapid dissolution rates and decomposition). For instance, the cytotoxicity of strontium-doped CaP coatings deposited onto AZ31 degradable magnesium alloy was evaluated with MC3T3-E1 mouse preosteoblasts and human mesenchymal stem cells (hMSCs), for contact times of 24–72 h [29]. For both cell types, the proliferation decreased upon increasing the Sr concentration. However, both osteogenic gene and protein expression significantly increased upon increasing Sr concentration. These results suggest that Sr-doped coatings are capable of promoting osteogenesis, in comparison to the undoped CaP coatings [44]. 

Generally, the safety assessment is clearly outlined by regulatory documents (such as the 10,993 series), while the efficacy assessment is more prone to the application of different testing methods of choice, involving both in vitro and in vivo assays similar to the situation for efficacy testing of medicines. 

The most relevant colorimetric method for evaluating cytotoxicity is the neutral red (NRU) assay, based on the ability of viable cells to incorporate and retain neutral red dye in lysosomes [45]. Comparing NRU results obtained for HA, natural coral, and polyhydroxybutarate on CRL-1543 cells, Shamsuria et al. concluded that all materials are non-cytotoxic even after 72 h of treatment [46]. Moreover, HA induced osteoblast cell proliferation (123% vs. control) which could be interpreted as biofunctionality [46]. However, NRU and MTT tests showed that HA-based cements can exert cytotoxicity by changing the concentration of ions (calcium, magnesium, and phosphate) in cell culture media [47]. Quantitative cell viability measurement using the NRU test allows identifying materials able to promote cell growth in regeneration studies. 

### 2.2. Cell Viability and Cytotoxicity

In most cases, HA materials are considered highly biocompatible and, therefore, suitable for bone tissue applications (e.g., medical implants and bone regeneration) [48,49,50]. However, the response of biological tissues and cell lines to HA-based materials (including HA nanoparticles and nano-objects) shows great variability [51,52,53,54]. 

Colorimetric viability tests are used for in vitro biocompatibility evaluation, where the absorbance of sample presumably is directly proportional to the number of viable cells [55]. Colorimetric viability tests using formazan salts (MTT, MTS, 2,3-bis-(2-methoxy-4-nitro-5-sulfophenyl)-2H-tetrazolium-5-carboxanilide (XTT), and Cell Counting Kit-8 (WST-8)) are based on the fact that mitochondrial activity and cell viability are correlated. Most common formazan salts used to assess cell viability are MTT (3-(4,5-dimethylthiazol-2-yl)-2,5-diphenyltetrazolium bromide), MTS (3-(4,5-dimethylthiazol-2-yl)-5-(3-carboxymethoxyphenyl)-2-(4-sulfophenyl)-2H-tetrazolium), XTT (2,3-bis-(2-methoxy-4-nitro-5-sulfophenyl)-2H-tetrazolium-5-carboxanilide), WST-1, and WST-8 reagents [56,57,58,59,60]. The MTT/MTS test is widely used to assess the biocompatibility of CaPs [52,61,62,63]. Using the MTS method, Santos et al. [52] showed that both chemically and hydrothermally synthesized HA did not affect the viability of MG63 cells treated for three and six days with HA concentrations lower than 500 μg/mL. Their results are important in the context of sterilization of HA-containing medical devices. Applying the MTT test on the same cell line (MG63 osteoblast-like cells), Aghaei et al. [64] demonstrated the biocompatibility of a silica mesoporous (MCM-48)/HA composite and its potential use as a drug delivery agent. The potential cytotoxicity of nanostructured HA was evaluated (MTS and LDH method), aiming to obtain highly biocompatible materials for bone surgery and dentistry applications [65,66].

In interpreting the data, it must be taken into account that an increase in the absorbance of samples may be due to an increased mitochondrial activity (with no significant change in the number of cells), rather than cellular proliferation. In addition, the number and activity of mitochondria in the tested cells are important, especially when discussing the comparative effect of materials on different cell lines or depending on the stage of cell differentiation [1,67]. In methods in which liquid biological samples containing the compounds of interest are subjected to optical absorbance determinations, the materials possessing a high refractive index, as well as fluorescence and photocatalytic properties, may induce altered results [68,69,70,71,72]. During optical density determinations, by absorbing or reflecting the incident light, suspended nanoparticles may increase the measured absorbance (turbidity/opacity). When exposed to ultraviolet (UV)–visible light, materials with photocatalytic properties may lead to the generation of reactive oxygen species and redox reactions on their surface, processes which may alter the molecules of interest in the biological sample or generate unknown or unwanted reaction products. Fluorescent nanomaterials may emit light at unwanted wavelengths which may induce erroneous results in optical determinations or induce undesirable physicochemical processes in the tested samples. 

Given that colorimetric viability tests (using both tetrazolium salts and LDH activity) are based on comparing the absorbance of the samples with the absorbance of the controls, and the optical density of the solution depends on its clarity, it is necessary to perform parallel non-cellular tests (i.e., by applying the exact same working protocol, but in the absence of cells). These should follow the exact same working protocol, but in the absence of cells. If the absorbance of the samples differs from that of the control samples, the values of the optical densities obtained in the cell test are subtracted from the values corresponding to the cell test.

Moreover, the protein corona effect has to be considered. Adsorption on the surface of bioceramic samples of molecules from the biological environment can have different biological effects compared to the initial materials, due to modified colloidal stability, changes in shape and volume, hiding of some functional groups, and exposure of cryptic peptide epitopes [73,74,75]. In vitro and in vivo protein corona-dependent changes in viability were already shown for HA and magnetic HA scaffolds [76,77]. Considering all error sources mentioned above, for the accurate evaluation of biocompatibility, it is recommended to use at least two different techniques (with respect to cellular mechanisms and/or principle of the technique). Biocompatibility studies require a good characterization of the tested materials concerning all aspects potentially involved in biological effects; the choice of testing methods should be done according to these specific characteristics of the bioceramic samples. The cellular viability and cytotoxicity in the presence of CaP-based bioceramics on several types of cells are summarized in Table 1.

### 2.3. Genotoxicity

Genotoxicity assessment represents an inevitable assay for permanent implantable bioceramics. It is done observing the regulation of ISO-10993-3:2014 “Test for Genotoxicity, Carcinogenicity, and Reproductive Toxicity” [78].

Both in vivo and in vitro methods can be used, but the recommended and largely applied genotoxicity assays rely on in vitro methods, due to the practical advantages (low amounts of compounds, ease of set-up, and compatibility to automation) and the circumvention of laboratory animal use. Several gene effects can be produced by genotoxins—gene mutations, chromosomal aberrations, and other DNA effects. No in vitro assay covers all possible effects. According to ISO 10993-3:2014, the use of in vivo methods is required only if in vitro testing indicates a need for further testing, due to possible pharmacokinetic mechanisms or complex metabolic effects leading to bio-activation of the compounds. Generally, the testing methods indicated in ISO 10993-3:2014 are further detailed in several Organization for Economic Co-operation and Development (OECD) guidelines [79]. Some modifications of the protocols are required, since the original methods were designed for soluble compounds (OECD guidelines); for biomaterials, modifications were made, such as accommodating the evaluation of fluid extracts. This is usually achieved by the use of fluids able to extract polar and non-polar chemicals, and the methods are described in ISO 10993-12:2012 “Sample Preparation and Reference Materials”. Depending on the test model used, the incorporation of nutrient media can also occur in the extractive formulation [80].

#### Gene Mutation Tests 

Such point mutations affect small portions of DNA molecule and include frameshifts and base-pair substitutions. The most common assay for the detection of such mutations is the Ames bacterial reverse mutation assay, using histidine-dependent *Salmonella typhimurium* strains as the test organisms. S9 active rat liver microsomes are also incorporated in the assay, to provide simulation of whole-animal exposure. There are several distinct strains (3–5), eliciting distinct mechanisms of DNA damage. Following exposure, the cells are reverted and regain the ability to grow without histidine, thus allowing them to be counted on the plates. 

A mammalian system used to detect gene mutation is the mouse lymphoma assay, using L5178Y cells [81]; these are exposed to extracts, with or without metabolic activation. After incubation, cultures are cloned in restrictive media for mutant phenotypes, and then assessed at the thymidine kinase (TK) locus to detect base-pair mutations, frameshift mutations, and small deletions. Cells that underwent mutations in the TK locus become resistant to growth in the presence of trifluorothymidine (TFT), unlike the parental cells, which cannot grow. Since mutant colonies exhibit a characteristic size distribution frequency, colony measurements can be used to distinguish the type of genetic effect.

Chromosomal aberration tests are used to detect chromosomal damage induced after one cellular division. The in vitro model employs Chinese hamster ovary cells. The assay is performed in the presence and absence of exogenous metabolic activation. Most aberrations can be identified as either chromosomal or chromatid type. Gaps, breaks, and exchanges are other examples of observable aberrations. 

More recently, a relatively rapid test, the Comet assay, which detects the amount of broken DNA (the tail length), was proposed. The assay can be achieved on any cell line, and it is relatively fast and reliable [82].

By using the Ames test and the Comet assay, Wahab et al. [83] evaluated the genotoxic risks following the exposure of dental pulp cells to biphasic calcium phosphate (BCP). The study revealed that the average number of revertant colonies in the Ames test was about half of the number of revertant colonies in the negative control plate, meaning that the compound did not display any genotoxic effect. 

Using a model of cultivated hepatocytes, Sonmez et al. [84] evaluated the several potential toxic and genotoxic effects of HA nanoparticles (NPs). With regard to genotoxicity, they evaluated the rate of the liver and measured the levels of 8-oxo-2-deoxyguanosine (8-OH-dG). Using increasing doses of NPs, they found increases in the number of micronucleated hepatocytes and 8-OH-dG levels compared to the control culture; however, these occurred only at high doses (1000 µg/cm^2^). 

Coelho et al. [41] investigated both cytotoxic and genotoxic effects of a bacterial cellulose membrane functionalized with HA and bone morphogenetic protein (BMP). Genotoxicity was evaluated by applying the in vitro Comet and micronucleus (cytokinesis-blocked micronucleus) assays on C3T3-E1 cells. The findings demonstrated that bacterial cellulose–HA was not genotoxic compared with the negative control, in both testing models. 

Seyedmadiji et al. [85] investigated the functionality of HA/bioactive glass (BG) and fluorapatite (FA)/BG materials. They also employed the Comet assay to investigate potential genotoxic effects on Saos-2 cells and found a dose-dependent increase in DNA degradation, but within the limits of safety (therefore, below any threshold of genotoxicity). Kido et al. [86] used the Comet assay as a final assessment for genotoxicity on tissue samples obtained from rats that were exposed to a ceramic scaffold covered with HA and bioglass; their assays demonstrated the lack of genotoxic effects of the investigated material. Oledzka et al. [87] investigated the cyto- and geno-toxicity of a new multifunctional composite based on HA porous granules doped with selenite ions (SeO_3_)^2−^, and their study proved that the investigated materials were non-gentotoxic, as demonstrated by the Umu test (carried out on *S. typhimurium* TA1535/pSK1002). Yamamura et al. used in vivo models for the investigation of biocompatibility, and the lack of cyto- and geno-toxicity in blood, liver, kidney, and lung was noted 30 days after HA implantation [88].

Several studies [86,87,88] generally demonstrated, using various models, the lack of genotoxicity of HA-based materials.

### 2.4. Oxidative Stress

A critical issue in regenerative medicine and tissue engineering (bone engineering included) is related to oxidative stress and altered redox signaling, which may be inflicted on normal and pathologic cells by the implant itself as an unwanted side-effect [89]. Oxygen is critical for aerobic organisms but it is also the source of reactive oxygen species (ROS), such as superoxide anion, hydrogen peroxide, and hydroxyl radical [90,91]. Low levels of ROS are necessary for physiological cell functioning by modulating cell survival and differentiation through tightly regulated redox signaling pathways [92]. Meanwhile, if ROS levels overcome a cell-dependent threshold through increased ROS production and/or downregulation of the endogenous antioxidant system [93], a deleterious oxidative stress is generated, which can seriously alter proteins, lipids, carbohydrates, and nucleic acids, thereby profoundly disturbing cellular homeostasis and even cell survival [90]. Persistent deregulation of the redox balance (antioxidants versus oxidants) may have long-term consequences on tissue physiology [94], thus raising concerns regarding the safety of biomaterials, including bioceramics.

Considering the critical involvement of ROS in many physiologic and pathological processes, a large panel of methods was developed for precisely detecting various types of ROS [95]. Current technical challenges are mainly related to the short life of highly reactive species. Conventional methods for detecting ROS rely on their ability to change the ROS indicator and to shift it to a more stable oxidized form. Nevertheless, the specificity of the currently available indicators for particular types of ROS is poor, and sometimes the chemistry behind the detection method is not very well characterized and, therefore, data may be misinterpreted. A more precise method to detect ROS with a radical nature (e.g., superoxide anion and hydroxyl radical) is electron paramagnetic resonance (EPR) with specific spin traps such as cyclic nitrones that form relatively stable spin adducts with radicals with a longer half-life to allow detection [96]. The EPR method is not easily accessible for most biomedical laboratories due to the high cost of the equipment and the required expertise to process EPR data. Assessment of oxidative stress markers in biologic fluids and tissues might be more informative for an initial screening aimed at evaluating the magnitude of the oxidative stress [97]. Only afterward is it worth attempting to define the ROS profile and dynamics for in-depth mechanistic studies. The in vitro studies performed so far regarding the impact of HA on ROS generation by various types of cells were performed by flow cytometry using the general intracellular ROS indicator dichloro-dihydro-fluorescein diacetate (DCFH-DA) [98] or by luminol-enhanced chemiluminescence for detecting released ROS [99], as described below. 

Considering that specific groups on the surface of functionalized HA-based implants may be highly reactive sites that interact with molecular dioxygen and generate uncontrolled oxidative stress, ROS-induced cytotoxicity is becoming an important component of the screening panel for assessing biocompatibility. Almost all studies developed so far for evaluating in vitro the impact of HA on oxidative stress were generally performed using HA NPs, and this was mainly due to methodological drawbacks in assessing the oxidative activity of cells cultivated on discs that better mimic the implant surface than NPs. Nevertheless, HA NPs covering orthopedic, spinal, and dental implants made of metals, ceramics, and polymers are under investigation, aiming to improve their osseointegration through enhanced biomimicry of host structures [100]. Moreover, HA scaffolds, alone or combined with bioactive molecules or genes and cells, are now being tested as promising bone grafts in hard-tissue engineering [101,102]. 

Oxidative stress and inflammation represent the best developed paradigm to explain many of the toxic effects of NPs in general [103]. The main mechanisms through which NPs can trigger enhanced oxidative stress comprise [104] (a) pro-oxidant or active redox cycling functional groups on the surface of NPs, (b) particle localization in cellular compartments involved in ROS generation, such as the electron transport chain in mitochondria [105] or activation of reduced membrane nicotinamide adenine dinucleotide phosphate (NADPH) oxidases [106], resulting in increased superoxide formation and further generation of more aggressive types of ROS (hydrogen peroxide and hydroxyl radical) through enzymatic reactions mediated by superoxide dismutases (SOD) or chemical reactions (Haber–Weiss and Fenton-type reactions) [107], and (c) indirect generation of ROS through inflammatory responses elicited by NPs, that are mediated by upregulation of NF-κB (nuclear factor κB), mitogen-activated protein kinase (MAPK), and phosphoinositide 3-kinase (PI3K) signaling pathways [108,109]. 

Due to its chemical and structural similarity with bone mineral, HA exhibits good biocompatibility, non-immunogenicity, and high osteoinductivity [110]. Early investigations on the biocompatibility of HA particles of different sizes, performed using bone-relevant cells (primary osteoblastic/osteoclastic cells), evidenced that larger-sized microparticles (500–841 μm) presented a better biocompatibility profile with respect to smaller-sized ones (<53 μm), which were shown to promote osteoclast activity and to restrain osteoblast activity [111]. As such, damaging effects are expected in the case of implant fracture or abrasion/erosion. It was also found that the uptake of nano-sized HA (20–80 nm in diameter) by bone marrow mesenchymal stem cells (MSCs) and osteosarcoma cells (U2OS) was dependent on the size of NPs and the type of cells [112]. Thus, 20-nm-sized HA particles greatly sustained the proliferation of beneficent MSCs, while the multiplication of tumor cells was inhibited. It is worth mentioning that human adipose-derived stem cells (hADSCs) which exhibit higher capacity of proliferation and multi-lineage differentiation in vitro are considered the most attractive MSCs due to the ease of their withdrawal and large availability. hADSCs can be charged in HA scaffolds, which promote in vitro osteogenic differentiation [113] and ectopic bone formation using HA scaffolds subcutaneously implanted in mice [114]. An increase in mitochondrial metabolism and consequent ROS generation underline the adipogenic differentiation of MSCs, as demonstrated by specific inhibition of the mitochondrial respiratory pathways [115].

Various in vitro studies highlighted that the interaction of cells with HA NPs can be modulated not only by size, but also through their charge and shape, thereby influencing their biocompatibility and the intended medical use. It was demonstrated that the uptake of nano-sized HA with a positive charge by MC3T3-E1 mouse pre-osteoblastic cells was higher compared to HA NPs bearing a negative charge, possibly due to better interaction with the negatively charged cell membrane [116]. The shape of HA nanostructures was proven to influence cellular viability and proliferation [117]. Thus, the study of Xu et al. [118], using the MTT assay and primary cultured rat osteoblasts, demonstrated that HA nanostructures with smaller specific surface areas induced lower apoptosis rates in the concentration range of 20–100 µg/mL. Moreover, needle-shaped particles (10–20 nm in diameter and 30–50 nm in length) inflicted greater cellular injury than spherical (10–30 nm in diameter) or rod-like particles (20–40 nm in diameter and 200–400 nm in length; 20–40 nm in diameter and 70–90 nm in length). At higher concentrations (100 µg/mL), the mentioned nano-sized HA particles were shown to induce enhanced ROS generation, as assessed by flow cytometry with DCFH-DA. The most active were the needle-shaped and spherical NPs which also induced higher apoptosis rates, thus indicating that oxidative stress underlined at least partially the observed cell death. Jin et al. [119] evidenced that HA nano-rods (~20 nm in diameter and ~80 nm in length) in the concentration range of 10–40 µg/mL were taken up into MC3T3-E1 mouse pre-osteoblasts via micropinocytosis and induced apoptosis through mitochondria- and lysosome-dependent damage pathways (mitochondria: altered expressions of B-cell lymphoma 2 (Bcl-2) and Bcl-2-associated X protein (Bax), decrease in mitochondrial membrane potential, and activation of caspase 3; lysosomes: lysosomal membrane permeabilization and increased the release of cathepsins B). Apoptosis was mainly correlated with oxidative stress ensuing from increased ROS formation (assessed by flow cytometry with DCFH-DA) and a decrease in endogenous antioxidant mechanisms related to SOD and glutathione peroxidase (GPx) activities. The involvement of this redox shift in inducing cell death was demonstrated using the ROS scavenger N-acetylcysteine (NAC) which was able to significantly protect osteoblasts against the apoptotic signals delivered by oxidative stress. 

The clinical failure or success of an implanted bioceramic depends not only on the bone remodeling cellular system comprising osteoblasts, osteocytes, and osteoclasts, but also on the interaction of blood leukocytes with the implant surface and the released ionic components. Phagocytes (monocytes, macrophages, and neutrophils) mediate key events for tissue repair at the interface with the implant surface, and inflammation triggered by these cells is essential for promoting wound healing and for restoring local homeostasis [120]. The pro-inflammatory activity of monocytes/macrophages can be modulated by the surface topography of HA-based implants [121]. For instance, monocytes cultivated on HA discs with plate-like surface morphology (micrometric size) were shown to release higher levels of ROS (detected using a luminol-amplified luminescence assay) as compared to monocytes cultivated on discs made of needle-like agglomerates (nano-sized); thus better inducing bone healing [122]. Therefore, a smart design of implant surfaces is needed for improving the clinical performance of HA-based implants through modulation of inflammatory and oxidative processes which greatly impact bone remodeling.

HA NPs also impact angiogenesis, an important mechanism involved in tissue repair and regeneration, aimed at fulfilling the increased local need for nutrients, oxygen, and growth factors [123]. As comprehensively demonstrated by Shi et al. using umbilical vein endothelial cells (HUVECs) [28], HA NPs (~20 nm and ~80 nm) were taken up by endothelial cells via energy-dependent endocytosis pathways involving clathrin and caveolin, while micro-sized NPs (~12 µm) were incorporated by macrocytosis. HA NPs localized and interacted mainly with lysosomes, and induced a decline in nitric oxide (NO) production in a concentration-dependent manner, in line with the trends seen in cell migration and tube formation. The major impact of HA NPs on endothelial cells seems to be underlined by the downregulation of reactive nitrogen species (RNS), and not by enhanced ROS production. The reduction of NO levels was attributable to a dose-dependent decrease in the phosphorylation of endothelial nitric oxide synthase (eNOS), which was associated with the downregulation of phosphorylated protein kinase B (Akt). These alterations in the PI3K/Akt/eNOS signaling pathway trigger a decrease in the viability of endothelial cells and tube formation ability, thereby limiting angiogenesis and tissue repair. Moreover, PI3K/Akt inhibition is expected to hinder the activation of NADPH oxidase and, hence, limit superoxide-driven oxidative stress in endothelial cells [124]. 

HA was also investigated in relation to tumor cells considering its potential applications in tumor-associated bone segmental defects [125]. Han et al. [126] demonstrated using cancer (MGC-803, Os-732, Bel-7402) and normal cell lines (L-02 hepatocytes, MRC-5 lung fibroblasts and HaCaT keratinocytes) that HA NPs, especially those with lower dimensions of 60 nm, inhibited the proliferation of tumor cells more than normal ones, partly due to enhanced incorporation. This effect was also attributed to a decrease in protein synthesis following the interaction of ribosomes with NPs, and was apparently not related to increased levels of ROS. Other studies also emphasized that HA NPs may specifically kill or inhibit the proliferation of tumor cells, such as osteosarcoma cells [127]. The cytotoxic effect exerted by HA NPs in cancer cells was not underlined by an enhanced oxidative stress, as stated by the authors, considering that the activities of succinate dehydrogenase and SOD, key enzymes responsible for ROS generation and scavenging, respectively, were significantly decreased by HA NPs in both cancer and normal cells [126]. Different results were communicated in the study of Xu et al. [128], showing that the observed decrease in the number of human gastric C6 cancer cells exposed to HA NPs was due to apoptosis associated with increased ROS generation (detected by flow cytometry with DCFH-DA) and decreased antioxidant defense mechanisms related to SOD activity. Pre-treatment of cells with the ROS scavenger N-(2-mercaptopropionyl)-glycine was able to protect glioma cells against the HA-triggered apoptosis, thus demonstrating that increased ROS levels indeed contributed to cell death. The conflicting results obtained in different studies might be attributed to distinct reactivity to HA treatment exhibited by different types of cells with various origins and status (normal or tumor), or might be related to dosage, size, or shape of the investigated NPs. For instance, Zhao et al. [129] showed that rod-shaped HA NPs with different surface areas, in the concentration range of 10–300 μg/mL, did not affect the viability human epithelial virus-transformed lung cells (BEAS-2B), murine macrophages (RAW264.7) and human hepatocellular carcinoma cells (HepG2). Nevertheless, a significant, but not cytotoxic, increase in ROS generation was registered at 4 h post exposure, as detected by flow cytometry with DCFH-DA. The highest levels of ROS were generated by those NPs having the largest specific surface area, which increased the cell–particle interaction. 

Altogether, there is experimental evidence that HA NPs, in particular situations, may induce oxidative stress in cells involved in osseointegration of implants and in bone remodeling. Accordingly, there is an urgent need to identify and develop therapeutic strategies aimed at limiting such alterations of the redox balance that may compromise the clinical outcomes of implantation. 

Doping of HA with cerium recently emerged as a promising approach in bone implantation, providing a complex control of oxidative stress and redox signaling through the redox cycling of cerium ions [130,131]. Cerium NPs (ceria) are used for their remarkable antioxidant activities [132], deriving from the changes in the oxidation state between Ce^4+^ (fully oxidized) and Ce^3+^ (fully reduced) and from the exquisite ability of cerium to adjust the electronic configuration for best fitting the close environment and for recycling [133]. Cerium is a potent ROS scavenger which significantly decreases the levels of ROS, especially of the toxic hydroxyl radical [134]. Moreover, it exhibits excellent SOD and catalase-mimetic properties, thereby reinforcing the cellular tools for antioxidant protection against the bursts of superoxide anion and hydrogen peroxide at the cell–implant interface. It was found that higher levels of Ce^3+^ were efficient scavengers of superoxide [135], while higher levels of Ce^4+^ favored the catalase-mimetic activity [136]. The Ce^3+^/Ce^4+^ oxidation state ratio was shown to be dependent on the primary particle size, with smaller cerium NPs having increased concentrations of Ce^3+^ as compared to their larger counterparts [137]. Moreover, cerium oxide proved good oxidative-stress anti-microbial activity [138], especially against Gram-negative bacteria. Therefore, cerium provides an additional advantage by counteracting potential low-grade infections at the site of implantation, thus reducing the need for prophylactic antibiotic treatment.

From the redox perspective, another option for doping HA is to use selenium, an essential micronutrient involved in various metabolic processes [139] as part of selenoenzymes, which are able to prevent and reverse even severe oxidative damages [140]. Selenium was also shown to play an important role in bone development [141]. The selenite ion represents a physiologic ionic doping agent for HA due to its almost identical size with respect to the orthophosphate tetrahedron. As demonstrated by Uskoković et al. [142], nano-sized selenite-HA has osteoinductive effects by eliciting an overall higher metabolic activity of cells. It is worth noting that, although selenium has antioxidant functions, selenite itself may trigger ROS production at levels that sustain osteogenesis [143]. Only if ROS levels increase above a threshold, for instance, in the case of cells exposed to NPs containing 3% selenite, do the apoptotic pathways get activated and cell death occurs [142]. The cytotoxic effect of selenite is of paramount importance for inhibiting osteoclastic bone resorption [144], as well as for decreasing the progression of certain bone tumors [144]. Selenite-induced oxidative stress and consequent apoptosis were found to be closely related to the intracellular level of reduced glutathione (GSH), a major cellular antioxidant molecule, with a key role in bone remodeling [145]. GSH proved to have a dual role in the effects of selenium on cancer versus normal cells. Thus, GSH may act as a pro-oxidant in cancer cells, facilitating selenium-induced oxidative stress, and as an antioxidant in normal cells, protecting them against oxidative stress and apoptosis. In addition to these effects, selenite-doped HA, like cerium-doped HA, exhibits anti-microbial activity [142] and could, therefore, reduce the post-implantation use of antibiotics. Altogether, selenite–HA materials show characteristics that make them suitable for improved osseointegration of bone implants and also for counteracting potential side-effects related to oxidative stress and infection.

A more general therapeutic approach to control the redox balance might be the pharmacologic activation of the cytoprotective transcription factor nuclear factor erythroid 2-related factor 2 (NRF2), which boosts the endogenous antioxidant system and controls the transcription of more than 250 cytoprotective genes [146,147]. Moreover, NRF2 was shown to contribute directly to bone remodeling by maintaining the equilibrium between osteoblast and osteoclast activities [148]. 

Concluding, potential alterations of the redox balance by HA and/or by the doping agents has to be taken in consideration when designing materials for bone implants, being aware that the levels of ROS and of endogenous antioxidant activity are critically involved in maintaining bone homeostasis and in sustaining regenerative processes.

### 2.5. Methods to Assess Oxidative Stress

Considering the critical involvement of ROS in various physiologic and pathological processes, a large panel of methods was developed and is still under construction for precisely detecting various types of ROS and their end result. More complex investigations regarding oxidative stress and redox signaling are needed for in-depth evaluation of the involvement of oxidative stress and redox signaling in bone regeneration and engineering using HA-based implants in order to identify therapeutic targets for improving osseointegration and the long-term outcome of implantation. 

## 3. Efficacy Evaluation of CaP-Based Bioceramics

**Figure 2:**
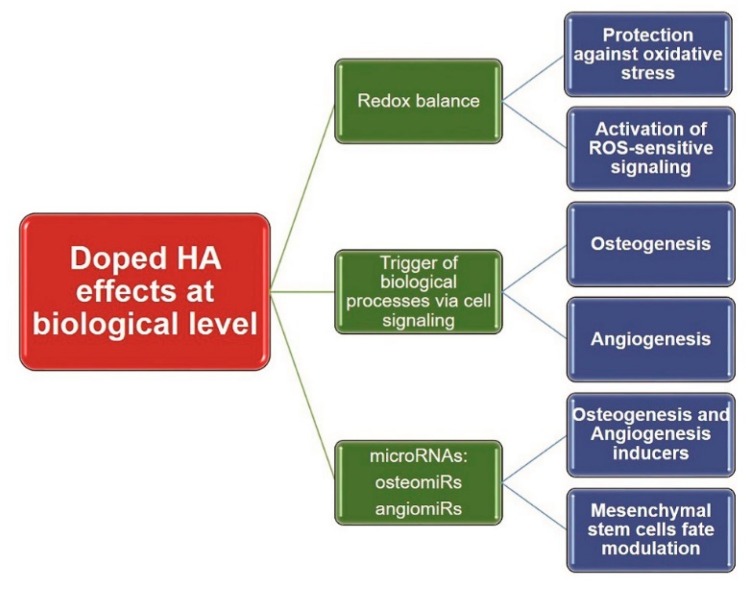
Interactions of doped hydroxyapatite (HA) with several main biological processes, and their cellular outputs.

CaP-based bioceramics have a demonstrated impact on various biological processes, such as redox balance, cell signaling, or epigenetic control of cellular activity, and various basic research findings of these interactions are discussed below. Figure 2 summarizes this interactions and cellular outputs, with a focus on doped HA.

### 3.1. Assessment of Osteogenic Effects

HA and its cation- and/or anion-substituted varieties are mainly envisioned for the development of bone regeneration applications. For osteogenesis studies, HA is used as implant coating, granules, or block bulk structures which act as non-resorbable materials in the short term, but still subjected to partial degradation and metabolization over time [149]. However, in addition to having poor mechanical properties, scaffolds of pure HA do not significantly promote vascularization and osteoinductivity [17]. Hence, additional doping is usually performed to augment osteoregeneration. HA can be modified by cationic and anionic substitution [1,19,150], or incorporated in a large variety of composite materials (together with metals, polymers, or proteins) [151]. The HA-based materials are evaluated on target cell populations and tissues (bone tissue and osteoblasts, alveolar bone, and tooth and mesenchymal cells), followed by animal testing. Although HA is a biocompatible material, each new composite or material containing HA must be tested again for cytotoxicity and biocompatibility, according to ISO 10993-6:2016 “Tests for Local Effects after Implantation”. For in vivo testing, an implantation test is the recommended method to evaluate the biocompatibility of a material with the surrounding tissue. Usually, intramuscular or subcutaneous implantation is routinely performed, but special sites, such as bone, can be used, if justified accordingly. Rabbits or small rodents are recommended, but larger mammals can be used for long-term testing, especially if the period of use exceeds the lifespan of a small rodent. For non-absorbable materials, such as HA, the short-term responses are normally assessed from one week up to 12 weeks, and the long-term responses are tested for periods longer than 12 weeks. Implantation in bone tissue may require longer observation periods before a steady state is reached. At the end of the testing period, histological sampling is used to assess the local tissue response to the implant [152].

Section 3.2 presents the most common in vitro and in vivo models for osteoregeneration studies of HA-based bioceramics.

### 3.2. In Vitro Models of Osteogenesis

In vitro models of osteogenesis rely on using osteogenic cell cultures, namely, osteoblasts, osteoblast-like cells (e.g., MG63 cells, which are derived from osteosarcoma, and MC3T3-E1), or MSC-derived osteoblasts. MSC-derived osteoblasts are obtained through special cell culture conditions, during which specific osteoblast differentiating medium is used, for a variable time length, depending on the protocol. MSCs can be derived from different sources, such as adipose [153], bone marrow [153,154,155,156], and umbilical cord blood [157]. Despite exhibiting significant similarities, MSCs from different sources are not identical in terms of osteogenic properties and response to osteogenic stimulation [153], which highlights the importance of selecting the appropriate combination of MSC population and stimulation for performing osteogenesis studies.

The effects of HA on osteogenesis are assessed by a number of methods. The most basic approaches measure the cell proliferation, which can be achieved through different techniques, from cell counting to double-stranded DNA (dsDNA) quantification, mitochondrial activity assessment, and real-time cell analysis [158]. These methods overlap with those used for assessing cytocompatibility/cytotoxicity and are covered more extensively in Section 2.2. However, osteoblast proliferation is merely a first step in studying osteogenesis in vitro. Analyzing cell proliferation alone is not sufficient for assessing osteogenesis, as, during this process, osteoblasts reach complete differentiation and stop proliferating, making functional readouts compulsory. 

Calcium deposition is indicative of complete differentiation of osteoblasts, and the Alizarin red S assay, based on a dye that binds calcium salts, was used to assess osteogenesis for decades [159]. However, this approach has limitations when it comes to studying osteogenesis using cells grown on an opaque substrate, as it does not allow for bright-field microscopy observation and subsequent quantification. Another method to assess the formation of new bone matrix that was used extensively is the von Kossa staining, a method that quantifies mineralization in cell culture, as well as tissue sections. However, this method is not specific to calcium phosphate; von Kossa staining alone was shown not to be appropriate for the identification and quantitation of bone-like mineral depositions, and it should only be used in combination with other methods to verify calcium phosphate presence and quality [160].

Alkaline phosphatase (ALP) is considered an early marker of osteoblast differentiation and represents a key player in mineralization [161,162,163]. ALP activity is frequently determined to assess functional osteogenesis using a *p*-nitrophenol phosphate disodium solution or commercially available enzymatic assays.

During osteogenesis, following the MSC proliferation phase, there is a significant expression of Runt-related transcription factor 2 (RUNX2), and its regulation is essential in bone formation [164,165,166]. The transcription factor RUNX2 has the capacity to upregulate the expression of collagen (Col-I), alkaline phosphatase (ALP), and osteocalcin (OCN) genes [167]. Moreover, the morphological changes and transformation of preosteoblasts into mature osteoblasts requires increased expression of (Osx) and secretion of bone matrix proteins such as OCN, bone sialoprotein (BSP) I/II, and Col-I [166,168,169,170,171]. Transcriptomic, proteomic, and metabolomic approaches, as well as immunodetection techniques (immunofluorescence - IFA, Western blot, enzyme-linked immunosorbent assay - ELISA), can be used to assess the expression levels of these osteogenic markers. Klontzas et al. compared the efficiency of different osteogenic agents on umbilical cord blood MSCs to promote the osteogenic process by quantifying osteonectin, OCN, ALP, and RUNX2 through qRT-PCR and reported differences between two commonly used osteoinductive agents, dexamethasone and BMP-2 [157]. Kulanthaivel et al. reported the improved osteogenic properties of Co^2+^- and Mg^2+^-doped HA, as confocal micrographs revealed increased expression of RUNX2 in MG-63 cells [172].

Transcriptomic, proteomic, and metabolomic approaches offer the ability to perform high-throughput studies; HTP proteomics includes both micro-arrays and mass spectrometry (MS) instruments; both are very sensitive, and both are able to generate a large amount of data. Microarrays are fast, with a large coverage of known proteins, and they could be useful if you want to “enlarge” the set of molecules to investigate. The same goes with MS instruments; the recent versions are faster and more sensitive, and they could be eventually used to both discover new markers and confirm the presence of some. 

Trabecular bone histological organization is characterized by a three-dimensional (3D) network of osseous trabeculae of calcified matrix and isolated mature osteocytes, creating a spongy scaffold. The cavities of this scaffold are lined by osteoblasts and filled with blood and blood cell precursors.

The compact bone is a highly 3D organized structure composed of osteons. An osteon is the structural and functional unit, comprising concentric bone cylinders, all centered by a canal outlined by osteoblasts and filled with blood vessels and nerves.

Existing 3D culture models for osteogenesis and/or bone tissue regeneration rely on scaffolds made of collagen-derived gelatin [173], collagen–HA [174], ceramic materials, inorganic HA [175], chitosan, and alginate [176,177]. These scaffolds are a better model for bone tissue. Curtin et al. showed that collagen–HA scaffolds have better osteoinductive properties when compared to collagen-only scaffolds, as revealed by Alizarin red and von Kossa stainings, results that corroborated well with osteocalcin expression, as shown by immunofluorescence staining [174].

While 3D models allow in vitro testing using structures that resemble bone tissue, reduced effects in 3D cultured MSCs in comparison to results in monolayers were noted in previous reports [156,178]. One study reported that the expression of OCN and ALP, as well as the activity of ALP, was reduced in a 3D model to approximately half with respect to that detected in a two-dimensional (2D) model. However, this reduction from 2D to 3D did not prevent the treatment from enhancing bone repair when tested in vivo [179], highlighting the importance of moving from in vitro models to in vivo ones.

In vivo HA testing is usually performed in rabbits or little rodent species over short-term periods of time. There are several animal models widely used for the assessment of HA substitutes on bone reconstruction (as reviewed in Reference [180]). For flat bone reconstruction, the most common is the calvarial defect model, which is frequently tested in small rodents: rat [181,182] and rabbits [183]. Long bone reconstruction uses a distraction osteogenesis model—a surgical model that creates a gap in a long bone (usually tibia), using continuous traction on the osteotomy ends. This model was used with various rodent species (e.g., mouse [184,185,186,187], rat [188,189,190,191], and rabbit [192,193,194]) and even larger mammalians (e.g., sheep [195,196] or goats [197]). Endo-osseous implants are used for dental reconstruction or oromaxilofacial reconstruction after tumor resection [198], but their biocompatibility also needs testing [199]. The evaluation of biological effects requires imagistic evaluation (radiographs, micro computed tomography (CT)), histologic evaluation of newly formed osseous tissue, and biomechanical testing. Depending on the need, there are also animal models for specific bone-related pathologies, such as osteoporosis (as reviewed in References [200,201]), osteonecrosis [202], or rare bone diseases [203].

It is now common knowledge that HA bioceramics or even HA-based composites are a suitable solution for osteoregeneration, from both mechanical and osteoinductive points of view. Due to improved fracture toughness, Mg- [27,204,205,206] and Zn-doped [207,208,209] HAs are most frequently tested for osteoregenerative properties; however, the incorporation of other ions was also tested. Lithium-doped HA scaffolds, for example, demonstrated improved mechanical properties and increased osteogenesis potential over pure HA [17]. The rationale of Li-doping is supported by several in vitro studies showing its role in osteogenic progeny growth and development, as well as in shifting mesenchymal stem cell fate toward osteogenic differentiation (as reviewed in References [1,210]). The main drawback of Li–HA is the lack of angiogenic stimulation [17,211], which can be overcome by including it in a composite [212]. Strontium doping was also favorable for osteogenesis when compared to pure HA in calvarial bone defect models [213], as well as in osteoporosis models [214,215,216]. Beneficial in vivo results were also reported for manganese, cobalt, copper, and gallium. For a more detailed presentation of different varieties of cation- and anion-substituted HA scaffolds and their biological effects, some recent reviews can be addressed [1,210].

The same animal models are used for testing whether the scaffold architecture matters or not for the biological output. Data from implant testing and bone-specific animal models showed that, beyond chemical composition, the osteoinductive properties of bioceramic-based bone graft substitutes depend on gross architecture (e.g., 3D printing versus nanostructured scaffolds [217], the thickness and size of macropores [218], or the nanoarchitecture of the scaffold [219]). Osteoregeneration and biomechanical properties are improved by tailoring of pore sizes [220] or even adjusting the shape and distribution of HA crystals [221]. Furthermore, Cu-doping was shown to be a modifier of the micro/nano-structured surface on the HA scaffolds, with a positive impact on angiogenesis [222].

As novel and better models emerge from preclinical testing, translation toward clinical implementation is also in need of standardization. HA derivates are used in clinical trials as substitutes for autologous bone grafts (for systematic reviews, see References [223,224]), and included in guidelines as grade C (low quality of evidence) [225]; hence, more clinical trials are required. Also, for standardization purposes, in 2016, the Special Interest Group on 3D Printing (SIG) was created to fulfill two goals: “to provide recommendations toward the consistent and safe production of 3D printed models derived from medical images, and to describe a set of clinical scenarios for 3D printing appropriate for the intended use of caring for patients with those medical conditions” [226]. In terms of bone reconstruction, so far only craniomaxillofacial reconstruction following trauma, genetic diseases, or different types of tumors was addressed [226]. The investigation methods used to assess osteogenic effects are summarized in Table 2.

### 3.3. Assessment of Angiogenic Effects

Mimicking the native microenvironment of bone extracellular matrix (ECM) by providing an optimal vascularization represents the major goal in bone regeneration processes, to be accomplished through a series of important characteristics: biocompatibility, optimal biodegradability, pre-vascularized structure, osteoconductivity, and less immunogenic responses. Unfortunately, currently available bioceramics do not entirely satisfy all these requirements [30,31,32]. The bone regeneration process is based on complex interconnected biological events including angiogenesis, osteogenesis, and inflammatory reactions [33]. 

Tissue regeneration is largely dependent on cell signaling that is mediated by cellular interactions with various key molecules, including growth factors. The angiogenic process involves a wide range of regulatory angiogenic proteins, such as vascular endothelial growth factor (VEGF), fibroblast growth factor-2 (FGF-2), epidermal growth factor (EGF), platelet-derived growth factor (PDGF), placental growth factor (PlGF), insulin-like growth factor (IGF), angiopoietins, matrix metalloproteinases, endostatin, thrombospondin-1, and insulin-like growth factor-binding protein-3, as well as pro-inflammatory cytokines (interleukin (IL)-1b, IL-6, tumor necrosis factor-α (TNFα)). The myriad of molecules involved in the regeneration processes are crucial players that trigger many signaling pathways, such as the VEGF signaling pathway, PI3K/Akt/eNOS, Ras/mitogen-activated protein kinase (MEK)/extracellular signal-regulated kinase (ERK), Sonic Hedgehog (SHH), Notch, and Wingless-related integration site (Wnt) [228]. 

Deficiencies in the vessel renewal or the paucity of neo-angiogenesis frequently result in delayed healing or tissue regeneration failure [229]. Notably, many studies reported in vivo implantation failures predominantly due to the lack of angiogenesis in the scaffold [230], delaying osteoid deposition and matrix development [229], consequently decreasing the bone healing rate [231]. Different strategies were designed to solve this issue, amongst which one can mention pre-vascularization by co-culturing of angiogenic and osteogenic cells [232]. 

To address these challenges, the HA-based bone scaffolds need to be designed for regulating cell behavior; therefore, generating the necessary angiogenesis is highly important for promoting a successful regenerating process.

A recent study pointed out that the expression of angiogenic genes (*eNOS*, *VEGF*, and *bFGF*) was upregulated in the conditioned media of HA micro/nanostructures, sustaining the high potential for inducing angiogenesis by regulating the immune microenvironment of macrophages. Based on these results, they pointed out that the basic regulation of macrophages influences the angiogenesis potential of endothelial cells (ECs), indicating that the biomaterial structure could regulate angiogenesis during tissue regeneration via a multi-pathway mechanism, either directly (endothelial cell stimulation) or indirectly (stimulation by activating macrophages) [233]. 

Aptamer-related technologies represent a revolutionary tool in bone tissue engineering. In this regard, a study conducted by Son et al. [234] developed an aptamer-conjugated HA (Apt-HA) that promotes angiogenesis, specifically targeting VEGF. In order to evaluate in vivo angiogenesis and bone regeneration, Apt-HA and HA were bilaterally implanted into a rabbit model and analyzed after eight weeks using micro-CT, histology, and histomorphometry. The results of this study demonstrated that Apt-HA showed significant increased blood vessel formation compared to simple HA, making the engineered Apt-HA an innovative candidate with great potential in promoting angiogenesis and bone regeneration [234]. 

Various potential strategies were explored to enhance the angiogenic capacity of the HA scaffolds. In this regard, several ions were observed to possess the potential to increase neovascularization (Mg, Cu, and Co), osteogenesis (Li, Zn, Sr, and Mn), or both (Si and B). 

Magnesium is known to be a fundamental element in bone and tooth composition, and it was already reported that increased levels of Mg were correlated with a favorable endothelial function. It was shown that high concentrations of Mg modulate in vitro vascular endothelial cell behavior, giving new insights into the role of Mg in angiogenesis. In addition, it was shown that high concentrations of Mg enhanced the synthesis of nitric oxide, through the upregulation of endothelial nitric oxide synthase (eNOS). In such a supportive microenvironment, endothelial cells are induced to migrate and grow, thus accelerating the formation of new vessels through a VEGF-like mechanism of inducing angiogenesis [235]. 

In this scenario, Deng et al. [13] studied the angiogenic effects in a goat model using magnesium-doped porous HA (MgHA) combined with recombinant human bone morphogenetic protein-2 (rhBMP2). The in vitro studies revealed that the VEGF expression, assessed by ELISA, for the 5% MgHA/rhBMP2 group was significant different compared with other groups at days seven and 14. The outcome was higher for the 5% MgHA group at day 14 than that of the other two groups. The in vivo study showed a significantly higher expression of VEGF and collagen I messenger RNA (mRNA) expression (evaluated by PCR) at 12 weeks in the MgHA/rhBMP-2 group.

Sartori et al. tested two new bi-layered scaffolds, one for chondral regeneration/type C (containing type I collagen), and another for the regeneration/osteogenic/type O of the subchondral layer (containing bioactive Mg-doped HA crystals), both seeded and incubated with hMSCs [236]. At four weeks from implantation in a mouse model, immunohistochemistry analysis revealed that only inside the type O scaffold layer was new vessel formation observed, suggesting a neo-angiogenesis process. Furthermore, a large number of positive cells for anti-human VEGF were observed from the scaffold surface to the center, while a few positive cells for anti-mouse VEGF were found near the scaffold surface. At eight weeks from implantation, bone tissue formation was observed in the O scaffold layer, appearing smooth and pale stained in comparison to the surrounding tissue. Osteoblasts were present on newly formed trabecular tissue using O scaffold as a template [236]. 

This study is in accordance with a Yang et al. report [237], which noted that the hMSC engineered 3D scaffold of HA/TCP, polyurethane (PU) foam, poly(lactic-co-glycolic acid)/poly(ε-caprolactone) electrospun fibers (PLGA/PCL), and collagen I gel seemed to be very effective in stimulating blood vessel formation, thereby ensuring facilitated oxygen and nourishment diffusion inside the scaffold, as also shown by the positive expression of anti-human VEGF by immunohistochemistry.

Canullo et al. [238], in a double-blinded randomized controlled trial, attempted to histologically evaluate the complex connection between angiogenesis and osteogenesis in post-extraction sockets enhanced with Mg-enriched HA, via indirect immunohistochemistry, using alkaline phosphatase, cluster of differentiation 34 (CD34), and caveolin-1 antibodies. The histomorphometric analysis indicated early angiogenesis followed by early osteogenesis processes, generated by Mg-enriched HA, which suggested it as a suitable material for post-extraction ridge preservation in dental medicine [238]. Also, a strong expression of caveolin-1 in preosteoblasts, osteoblasts, and osteoclasts was found [238], in accordance with Frank et al. [239], who noted that caveolin-1 was strongly abundant in endothelial cells regulating functions such as angiogenesis, vascular permeability, and transcytosis [239]. In another study on caveolin-1-deficient mice, angiogenesis was found to be markedly reduced in comparison with control mice [240].

Sun et al. [241] explored the functions and properties of an HA nanowire/magnesium silicate core–shell (HANW@MS/CS) porous scaffold by SEM (scanning electron microscopy) and inductively coupled plasma (ICP) optical emission spectrometry (for the release behavior of ions), and pointed out that HANW@MS/CS promoted the attachment and growth of rat bone marrow-derived mesenchymal stem cells (rBMSCs), inducing the expression of osteogenic differentiation related genes and the *VEGF* gene of rBMSCs. Moreover, the HANW@MS/CS scaffold stimulated in vivo angiogenesis and bone regeneration, by enhancing the gene expression of *VEGF*, *RUNX2*, *OCN*, and *OPN* (osteopontin) compared with the HANW/CS and CS scaffolds (assessed by RT-qPCR). The experimental results indicated that the abilities of these scaffolds in stimulating angiogenic and osteogenic responses of rBMSCs followed the trend HANW@MS/CS scaffold > HANW/CS scaffold > CS scaffold [241]. 

An interesting study designed by Calabrese et al. [242] attempted to evaluate the osteoinductive and angiogenic potential of a cell-free collagen–MHA (magnesium-enriched HA) scaffold containing a bilayer scaffold made of collagen I alone (layer 1: Coll) and collagen–MHA (layer 2: Coll–MHA), using innovative biomaterials that closely mimic the bone characteristics. Using fluorescence molecular tomography (FMT) imaging, an increase in de novo formation of vessels was revealed, and, using AngioSense 680, the fluorescent signal appeared reasonably elevated until four weeks, before recording a decreased signal by about 40%, with no statistical significance. Hematoxylin and eosin (HE) staining also confirmed the vascularization, indicating the presence of structures resembling blood vessels, which were abundant at four and eight weeks post implantation mainly within the Coll–MHA layer. The results revealed by HE staining were validated by the expression of CD31 endothelial marker, which recorded a weak signal at the first week, with an increasing trend up to week eight. These interesting findings bring novel insights into collagen–HA scaffolds designed using an innovative biological method, having the special capacity to recruit host cells by themselves, promoting bone regeneration. In addition, the spontaneous appearance of vascular structures within the innovative scaffold holds promise for successful bone regeneration using scaffolds alone, without supplementary growth factors or other in vitro manipulated cells, as many other studies confirmed [242]. 

Due to copper’s known stimulatory effect on endothelial cells toward angiogenesis, many studies were conducted using Cu-doped HA for increasing the angiogenesis capacity [243]. Barralet et al. discovered that a CaP scaffold doped with low doses of Cu^2+^ led to the formation of new vessels along the macro-pore axis, as confirmed by immunohistochemistry [244]. In addition, scaffolds containing angiogenic factors, especially copper and a copper–VEGF combination, expressed a greater degree of tissue ingrowth than the control. Moreover, the addition of Cu ions strengthened the bioactivity [222,245], in which it was shown that Cu-assisted hydrothermal deposition techniques provide a reliable route toward engineering micro/nano-structured surfaces on Cu-doped HA scaffolds, with beneficial properties in terms of angiogenesis and bone regeneration. Based on the findings that the doped elements not only affect the apatite physical structure, but also strengthen its biological function, it was observed, by scanning electronic microscopy (SEM), that Cu concentration also affects the morphologies of CaP coatings that grow on HA scaffolds, significantly increasing cell proliferation [222]. 

Strontium is currently used in the treatment of osteoporosis. Sr-doped HA scaffolds enhanced the solubility and stimulated earlier bone formation, while also sustaining a better cell attachment and proliferation [246].

Different in vitro studies mentioned that Sr-doped HA supports osteoblast proliferation and differentiation processes by triggering calcium-sensing receptors, as well as stimulating angiogenesis and osteogenesis. It was observed that, in comparison with calcium polyphosphate (CPP) and HA scaffolds, the formation of a tube-like structure and the expression of platelet endothelial cell adhesion molecule (PECAM) in the co-cultured model was better in the Sr-doped CPP (SCPP) scaffold [247]. Also, a positive effect of Sr on angiogenesis is supported by in vivo studies which revealed the formation of new vessels, highlighted by positive staining for CD31, especially in 50% (molar ratio) Sr-doped HA (50Sr-HA), after four weeks of implantation compared to HA and 8Sr-HA [248]. 

Also, the capability of Sr-doped calcium polyphosphate (SCPP) to stimulate angiogenic and osteogenic processes was analyzed in vitro and in vivo by Gu et al. [247]. They used an in vitro co-cultured model of human umbilical vein endothelial cells (HUVECs) and osteoblasts and then cultured the cells with SCPP, calcium polyphosphate (CPP), and HA scaffolds. Subsequently, ELISA analysis demonstrated that PECAM-1 concentration in the SCPP group was significantly higher than in the CPP group and HA group, with a maximum at the 28th day, in accordance with immunofluorescence analysis. Strings of tube-like structured (TLS) HUVECs were detected spreading through the co-cultured model. The PECAM-1 expression of HUVEC and the formation of TLS were longer for the SCPP group in comparison with CPP, demonstrating that SCPP has a higher ability to induce angiogenesis in vitro. The in vivo model revealed a positive immunostaining for VEGF in newly regenerated tissue in both CPP and SCPP groups at weeks eight and 16 post operation with more formation of new bone and tube-like structures (TLSs) in the SCPP group. On the other hand, at 16 weeks, the HA group presented a mild positive result in VEGF expression and the formation of bone, while no TLSs were observed. The intensity of positive VEGF staining decreased at 16 weeks in the CPP and SCPP groups. In conclusion, the SCPP scaffold could represent a potential biomaterial that stimulates angiogenesis in bone tissue engineering [247].

Cobalt represents another promising essential element in the bone regeneration field due to its hypoxia-mediated angiogenesis capacity by hypoxia-inducible factor (HIF-1α) activation [246]. Based on histological and immunohistochemical analyses, it was observed that the substitution of Co^2+^ could improve the angiogenesis properties of HA, by mimicking hypoxia conditions, upregulating HIF-1a and VEGF expression [249]. 

Dual doping of bivalent Mg^2+^ and Co^2+^ ions was evaluated, and the in vitro analysis on bone cells (MG-63) showed that HAC (5% (CoCl_2_MgCl_2_)–HA) induced the highest expression of VEGF, followed by HAN (5% (Co(NO_3_)_2_–Mg(NO_3_)_2_)–HA), while HA showed the lowest expression, equivalent to the control. This finding highlighted that the high expression of HIF-1α in HAC was directly influenced the VEGF synthesis. In brief, dual doping improves the osteogenic and angiogenic properties of HA, resulting in an improved biomaterial for bone tissue engineering [172]. 

Zinc is another essential trace element, being important in the structure of various metalloenzymes, such as alkaline phosphatase (ALP), an extremely important molecule for the maturation of new bone formation. 

Nano-HAs, with/without nano-zinc oxide (n-ZnO), were studied, and a chicken embryo chorioallantoic membrane (CAM) assay indicated the induction of angiogenesis for the scaffolds containing n-ZnO, as well as significant upregulation of angiogenic-related genes, confirmed by RT-PCR analysis. In consequence, scaffolds containing n-ZnO have substantial importance for inducing osteogenesis and angiogenesis processes in bone tissue engineering strategies [232]. 

Lithium is present in organisms as a trace metal, and various studies reported that Li could have effects in increasing bone density [250] and promoting osteogenic differentiation of bone marrow mesenchymal stem cells (BMMSCs) by activating the Wnt/glycogen synthase kinase 3β (GSK-3β) signaling pathway [251,252]. In a recent study, an innovative lithium-doped HA scaffold (Li-HA) was evaluated, seeded with hypoxia-preconditioned bone marrow mesenchymal stem cells (BMMSCs). When the seeded cells were preconditioned in hypoxia medium, the new bone formation was improved, with higher β-catenin and lower GSK-3β expression. Also, the HIF-1α, VEGF, and CD31 expression, evaluated by qPCR, was upregulated, exerting a positive effect on activating the Wnt and HIF-1α signaling pathway [211]. The investigation methods used to assess angiogenic effects are summarized in Table 3.

In conclusion, in bone tissue engineering, biological processes such as angiogenesis and osteogenesis are finely concerted during lifelong bone formation, and many studies established that the microenvironment could directly control the development of these processes.

### 3.4. Signaling Pathways Involved in Osteo- and Angio-genesis

**Figure 3:**
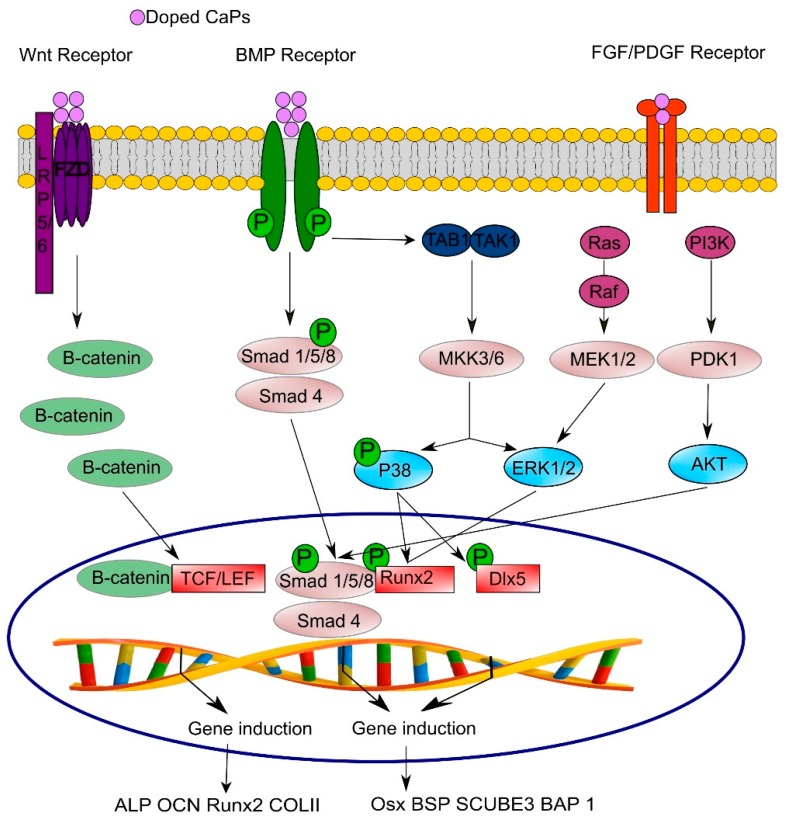
Cell signaling pathways activated in osteogenesis and angiogenesis models: Wingless-related integration site (Wnt) pathway, bone morphogenetic protein (BMP) pathway, and fibroblast growth factor (FGF)/platelet-derived growth factor (PDGF) pathway. Runt-related transcription factor 2 (RUNX2) is a major hub where all these pathways merge and cross-talk to guide the differentiation of bone and new vessels. Abbreviations: mitogen-activated protein kinase (MAPK), phosphatidylinositol 3-kinase (PI3K), osteocalcin (OCP), alkaline phosphatase (ALP), Osterix (Osx), BSP- Bone sialoprotein, BAP 1-BRCA1 associated protein-1, COL II- Collagen type II, SCUBE 3-Signal peptide-CUB-EGF-like domain-containing protein 3.

New bone formation, as well as bone regeneration and bone healing, requires both diffusible signals and proper vascularization. Hence, angiogenesis is frequently investigated alongside with osteoinductivity of various cation- and/or anion-substituted-HA bioceramics. Furthermore, the same signals (see Figure 3) are responsible for inducing osteodifferentiation/proliferation and angiogenesis, depending on the receiver cell type. 

Such extensive knowledge on signaling proteins involved in bone formation and regeneration was translated into clinical practice by clinical trials aiming at bone defect repair. To date, two members of the BMP family (BMP-2 and BMP-7) were approved as treatment in orthopedic and maxillofacial reconstruction. In selected pathologies, they were shown to outperform bone autograft, but potentially severe side effects called for caution in their clinical use [253,254,255]. Initially used as recombinant proteins, BMPs and growth factors are now delivered using various scaffolds, including HA substitutes [256,257,258]. HA by itself was shown to trigger “a specific intracellular signal transduction cascade during early osteoblast adhesion, activating proteins involved with cytoskeleton rearrangement, and induction of osteoblast differentiation” [259]. Additional growth factors such as VEGF, PDGF, IGF, or transforming growth factors (TGF-1 and TGF-2) can be adsorbed onto the bioceramic bone scaffold; however, to avoid the expensive costs, they were replaced with ions (e.g., Li, Co, Ni, Mg, Sr, and La) having similar effects. The incorporation of Au activates the Wnt/β-catenin signaling pathway, explaining the osteoinductive capability of HA–Au NPs [260]. The silk fibroin (SF)/HA/polyethyleneimine-functionalized graphene oxide (GO-PEI) scaffolds loaded with mir-214 inhibitor (SF/HA/GPM) increased the expression of activating transcription factor 4 (ATF4) and activated the Akt and ERK1/2 signaling pathways in mouse osteoblastic cells (MC3T3-E1) in vitro [261]. Boron-containing HA was shown to affect genes involved in Wnt, TGF-β, and response to stress signaling pathways [262]. An increase in CeO_2_ content in HA coatings increased alkaline phosphatase (ALP) activity, calcium deposition activity, and the Wnt/β-catenin signaling pathway [263]. HA promoted the osteogenic differentiation of hBMSCs, possibly by increasing cell attachment and promoting the Yes-associated protein (YAP)/Tafazzin (TAZ) signaling pathway [264].

The development of functional HA bound to signaling peptides for the promotion of bone regeneration was studied actively. A stimulating effect of bone cell growth by capturing VEGF from Apt-HA in both in vitro and in vivo environments was observed [234]. Thrombin-peptide 508 (TP-508), erythropoietin (EPO), and blocking of thrombospondin-2 (TSP2) could also improve bone healing via angiogenesis mechanisms [265]. For example, using HA-based scaffold of Li-nHA/ gelatin microsphere (GM)/rhEPO improved the viability of glucocorticoid-treated bone marrow mesenchymal stem cells and vascular endothelial cells and increased the expression of osteogenic and angiogenic factors. The Li-nHA/GM/rhEPO scaffold could upregulate the Wnt and HIF-1/VEGF pathways at the same time, with effects on improving osteogenesis and angiogenesis [212].

The VEGF-derived “QK” peptide was synthesized with a heptaglutamate (E7) domain, a motif that has strong affinity for CaP bone graft materials with greater activation of Akt and ERK1/2 in cells exposed to the E7–QK-coated discs. This angiogenic potential holds promise for augmenting the regenerative capacity of non-autologous bone grafts [266]. The connective tissue growth factor (CTGF)-loaded HA-based bioceramics could enhance cellular attachment through interaction with integrin, promoting actin cytoskeletal reorganization. CTGF-loaded HA also enhanced the differentiation of osteoblasts by integrin-mediated activation of the signaling pathways [267]. 

Improved vasculogenesis and bone matrix formation through a co-culture of endothelial cells and stem cells in tissue-specific methacryloyl gelatin-based hydrogels contributed to stimulate the interplay between osteogenesis and angiogenesis in vitro, as a basis for engineering vascularized bone [268].

Signals for osteo- and angio-genesis can also be delivered by means of the substrate micro-/nano-architecture. Bai et al. [269] demonstrated that nano-rod-decorated micro-surfaces better enable osteogenesis and angiogenesis, with respect to NP-decorated ones. Their study unraveled that the immune response of macrophages can be manipulated by the nano-/micro-surface, leading to a differential effect on osteointegration. The additional knowledge obtained from this study may provide a foundation and reference for the future design of coating materials for implantable materials [269]. 

Further understanding of cue signals that coordinate osteoinductive and pro-angiogenic effects will improve the generation of more performant HA-based bioceramic and biocomposite materials for orthopedic and dental applications.

### 3.5. MicroRNAs Involved in Osteo- and Angiogenesis

MicroRNAs (miRNAs) are evolutionarily conserved small non-coding RNAs, single-stranded molecules of about 22–25 nucleotides in length, which are involved in post-transcriptional gene regulation. MicroRNAs exert their function by partial or total binding to a specific mRNAs based on sequence complementary [270]. Series of miRNAs act in a complex functional network in which each miRNA might control hundreds of distinct target genes, and the expression of a single coding gene can be regulated by several miRNAs [271,272,273]. Up- or downregulation of the miRNA itself by stage- and tissue-specific expression patterns can lead to modified expression of its target genes and might be considered to act as fine-tuning of protein expression. 

In the past several years [274], major progress was made in understanding the biological functions of miRNAs in bone formation and remodeling. The development of the next-generation high-throughput sequencing technologies [275,276] made possible the identification of classes of microRNAs involved in osteogenesis and angiogenesis. The availability of synthetic enhancers (mimics) or inhibitors (antagomiRs) triggered the investigation of the potential of miRNA for improved biomimetic composites for smart materials, mainly in combination with bioceramics. Therefore, specific miRNAs could be exploited to either induce stem-cell chondrogenic differentiation for articular cartilage regeneration or osteogenic differentiation for bone regeneration [277]. 

OsteomiRs were identified to regulate chondrocyte, osteoblast, and osteoclast differentiation by positively targeting principal osteogenic transcription factors such as RUNX2, Osterix (Osx), and ATF4 (activating transcription factor 4), and several signaling pathways including BMP, Notch, and Wnt, which control osteogenesis [278,279,280]. For example, miR-31 modulates osteogenic differentiation and mineralization of hBM-MSCs, by targeting the bone-specific transcription factor Osx [281], and miR-20a controls the expression of other important proteins involved in osteogenesis—BMP2, BMP4, and RUNX2 [282]. There are also several microRNAs with a specific role in processes of osteo- and angiogenesis. The highly conserved microRNA, mir-9 positively stimulates osteo- and endothelial progenitor cell formation. The miR-9 mimic-transfected HUVEC cells showed increased VEGF, VE-cadherin, and FGF protein expression levels, leading to increased EC migration and capillary tube formation in vitro. The activation of the AMP-activated protein kinase (AMPK) signaling pathway was the underlying molecular mechanism for the regulation of osteoblast differentiation and angiogenesis [283]. Nevertheless, the exact mechanisms of skeletal miRNAs governing the complex interactions and signaling pathways of different bone-forming cells are only beginning to be elucidated.

To accelerate bone regeneration, cytokines and growth factors could be delivered at the implantation site, but their use in clinical settings is constrained due to the poor stability of proteins, high cost, and short half-life [284]. Thus, more proper alternatives are needed to accelerate bone formation, and microRNA delivery using biocompatible systems seems to be more appropriate and less expensive. 

For example, bone-specific miRNAs, such as miR-21 that promote osteogenesis in bone marrow stem cells, were delivered by biocompatible chitosan (CS)/hyaluronic acid NPs, thereby accelerating the osteogenesis process in human bone marrow mesenchymal stem cells (hBMMSCs) [285]. Also, the miR-21-functionalized microarc-oxidized (MAO) Ti surfaces demonstrated cell viability, cytotoxicity, and cell spreading comparable to that exhibited by naked MAO Ti surfaces and led to significantly higher expression of osteogenic genes. This novel miR-21-functionalized Ti implant may be used in the clinic to allow more effective and robust osteointegration [286].

Alternatively, the field of tissue engineering aims to regenerate damaged tissues, instead of replacing them, by developing biological substitutes that restore, maintain, or improve tissue function. The field relies extensively on the use of stem cells in combination with porous 3D scaffolds that house the cells and provide the appropriate environment for the regeneration of tissues and organs. The bioceramic-based HA NPs are potentially the main candidates as vectors, because of their major advantage of proven high biocompatibility. HA NPs offer additional advantageous properties for use in bone regeneration applications due to the chemical mimicry of the inorganic component of bones, as well as their demonstrated osteoconductive properties in vitro and in vivo [174].

In a recent in vitro study, a culture of stem cells derived from human periodontal ligament (hPDLSCs) was seeded on a scaffold made by fully deproteinated and sterilized HA bioceramic. The morphology, viability, osteogenic differentiation, VEGF release, and miR-210 expression of these cells were assessed. The promising results indicated that the 3D scaffold in contact with hPDLSCs showed good osteoconductive properties, evaluated through the adhesion and proliferation process, and presented the ability to stimulate VEGF secretion in hPDLSCs via miR-210 involvement. The induction of the production of this growth factor from hPDLSCs could represent a goal for tissue engineering, for the therapeutic growth of new blood vessels around the biomaterial in the first phase of osteointegration. Thus, the hPDLSC/glucose (G) construct could represent an interesting strategy to prefabricate a vascularized bone segment to be transplanted into the defect site [287]. 

Also, one study showed that the functionalization of porous collagen–nano-HA bone scaffolds with miR-133a-inhibiting complexes, delivered using non-viral HA NPs, enhanced human mesenchymal stem cell-mediated osteogenesis through the key activator of osteogenesis, RUNX2 [156]. The increased RUNX2 and osteocalcin expression, as well as higher ALP activity and calcium deposition, thus, demonstrated the further enhanced therapeutic potential of a biomaterial previously optimized for bone repair applications. In addition, miR-133a was identified as a direct negative regulator of the master transcription factor of osteogenesis, RUNX2; hence, the direct relationship between miR-133a levels and RUNX2 expression provides the possibility to target a central activator of osteogenesis. This nanoantagomiR-133a system also produced a rapid pro-osteogenic effect in hMSCs in 3D culture platforms [156]. The promising features of this platform offer the potential for applications beyond bone repair and tissue engineering, and constitute a new paradigm for microRNA-based therapeutics [156]. 

A study by Vimalraj et al. [288] demonstrated that a biocomposite scaffold based on carboxymethyl cellulose, zinc-doped nano-HA, and ascorbic acid (CMC/Zn-nHA/AC), along with microRNA-15b, transfected into mouse mesenchymal stem cells promoted osteoblast differentiation faster than control experiments. The early detection of alkaline phosphatase mRNA, which is an osteoblast differentiation marker gene [289], and the significantly increased expression of RUNX2 at the mRNA and protein level demonstrated the additive effect of the scaffold with bioactive molecule mirR-15b. This result demonstrated that biocompatible HA-based scaffolds might be functionalized with osteo-miRNAs in order to improve their response to osteogenic differentiation of mesenchymal stem cells. The increased effect of the bioengineered transfected cell-based scaffold suggested that there are different intracellular signaling pathways activated in cells, resulting in an enhanced osteogenic effect [288]. 

MicroRNAs are involved in several cellular mechanisms, but one distinct role of these non-coding RNA sequences is the modulation of the epigenetic mechanisms of gene expression. Epigenetic regulation is the biological mechanism whereby DNA, RNA, and proteins are chemically or structurally modified without changing their primary sequence. These epigenetic modifications play critical roles in the regulation of numerous cellular processes, including gene expression and DNA replication and recombination. Epigenetic regulatory mechanisms include, in addition to small (microRNAs) and long non-coding RNAs (lncRNAs), DNA methylation and hydroxymethylation, histone modification, chromatin remodeling, and RNA methylation. At present, there are a limited number of studies that investigated the impact of biomaterials on the epigenome, with most studies focusing on titanium and titanium dioxide (TiO_2_), and a few on silica, glass, and graphene [290,291].

It should be noted that these studies considered just the biomaterials of nanometer dimensions that can be absorbed into the cellular environment and that might have an immediate effect at the molecular level. A recent study reported the biological effects of nano-HA (10 nm up to 100 nm) on the lineage commitment and differentiation of bone-forming osteoblasts and highlighted the impact of HA on the epigenome [292]. The nano-HA stimulated a strong dose-dependent suppression of the ALP, BSP, and OSC RNA levels, and this effect was sustained for weeks even in the absence of nano-HA. The study reported a 40% increase in DNA methylation at the promoter region of the osteoblast lineage commitment alkaline phosphatase gene (*ALPL*) in murine bone marrow stromal cells, following treatment with nano-HA. In general, the gene’s promoter region hypermethylation is associated with gene silencing, and a less methylated promoter denotes a transcriptionally active gene. Furthermore, the exposure of osteoblasts to nano-HA resulted in dramatic and sustained changes in gene expression, whereas later-stage osteoblasts were much less responsive. These results suggested a potentially permanent alteration in the epigenome after HA exposure, with direct implication on osteogenic gene regulation. Collectively, this study identified for the first time that nano-HA is a potent regulator of the osteoblast lineage through changes in gene expression and identified methylation as a novel regulatory mechanism [292]. Although these results are interesting, future research analyzing single cytosine–phosphate–guanine (CpG) methylation at different regions of the *ALPL* gene is needed to determine the precise role of nanoparticle-dependent DNA methylation changes in gene expression and to determine the molecular mechanism through which nano-HA induces its effects [293]. 

Currently, there is limited knowledge on the epigenetic effects of biomaterials and the topography of 3D scaffolds on cellular activities. Greater investigations are necessary for a better understanding of the impact of biocompatible materials at the molecular level of the human epigenome. Finding the critical epigenetic mechanisms involved in stem-cell differentiation and studying the impact of bioactive material on the epigenome may be imperative for clinical translation into tissue engineering and bone regeneration.

## 4. Future Perspectives

Taken together, remarkable progress was made in unraveling the role of CaP-based bioceramics in stimulating angiogenic and osteogenic processes, which could open the path toward highly functional bone engineering medical devices with applicability in orthopedics and dentistry. Further studies are necessary for the in-depth evaluation of these complex processes by deciphering signaling pathways and miRNA involvement, using cutting-edge technologies for the assessment of the biological performance of such novel biomaterials and implants. The recent advancements in tissue engineering technologies, including three-dimensional (3D) printing, offers hitherto remarkable opportunities to develop a next generation of bone tissue substitute (grafts) with significant advantages over the conventional ones. Personalized implants can be produced with tailored characteristics better adapted to the patient-specific bone tissue regions/defects that are needed to be replaced/reinforced. To transfer these technologies to clinical practice, material science and tissue engineering need to be closely assisted by biomedical researchers in order to confer the safety risk assessment, as well as efficacy at high standards. A “systems biology” approach is needed for comprehensive analysis of the biological mechanisms of CaP-based bioceramics, with emphasis on the biocompatibility and biofunctional efficacy, joining critical processes such as oxidative stress angiogenesis, osteogenesis, etc. In the near future, the development of complex testing strategies will help to unveil the network of biological events elicited by CaP-based bioceramics in bulk, coating, or nanoparticle form, which are essential to ensure a longer and safer implant life in orthopedic and dentistry applications.

## Figures and Tables

**Table 1:** Cellular viability and cytotoxicity tests. CaP—calcium phosphate; HA—hydroxyapatite; MTS—3-(4,5-dimethylthiazol-2-yl)-5-(3-carboxymethoxyphenyl)-2-(4-sulfophenyl)-2*H*-tetrazolium; MTT—3-(4,5-dimethylthiazol-2-yl)-2,5-diphenyltetrazolium bromide; LDH—lactate dehydrogenase.

Type of CaP	Type of Cells	Methodological Approach	Main Effects	References
Cell Viability				
HA nanoparticles produced via wet chemical synthesis (37 °C) and hydrothermal synthesis (180 °C)	MG63 osteoblast-like cells	MTS cell proliferation assay	Neither particle, in doses lower than 0.5 mg/mL, affected cell viability and proliferation. For concentrations between 0.5 and 2 mg/mL, the inhibition of cell proliferation was time-dependent, with slightly higher values corresponding to chemically synthesized HA when compared with hydrothermally synthesized HA.	[52]
Nano-HA–silica-incorporated glass ionomer cement (HA–SiO_2_–GIC)	human Dental Pulp Stem Cells (DPSC)	MTT assay	HA–SiO_2_–GIC showed cytotoxic effects for all tested concentrations (3.125–200 mg/mL).	[61]
HA coatings prepared by a sol–gel method on Ti6Al4V	human fetal osteoblasts, subcultures 4–6	MTT assay	HA sol–gel-derived coatings showed low toxicity in osteoblast cell culture after 3 days (due to poor adhesion of the cells). Subsequently, cell viability increased in cells treated for 7 and 14 days with HA.	[62]
HA nanoparticles (HA NPs)	Reconstructed human gingival epithelium (HGE)	MTT test; LDH assay	3.1% HA NP solution did not induce cell death after 10 min, 1 h, and 3 h of incubation.	[63]
HA composite with the mesoporous silicate MCM-48	MG68 cells	MTT assay	MTT results showed the biocompatibility of the new material and supported its possible use as drug carrier.	[64]
HA–Au nanoparticles	Human mesenchymal stem cells	MTS test; LDH assay	When compared with controls, the MTS assay showed no significant differences in the cell viability of cells exposed to 1–100 μg/mL HA–Au nanoparticles. LDH results indicated minimal damage to the cell membranes.	[65]
High-temperature annealed nano-HA obtained via wet chemistry at 800 °C, 900 °C, and 1000 °C	L929 (NCTC clone 929) mouse fibroblast cells	MTT assay	All tested samples slightly decreased the viability of cells treated with 2.5, 5, 10, and 20 g/mL nanoparticle suspensions.	[66]

**Table 2:** Investigation methods used to assess osteogenic effects. MSC—mesenchymal stem cell; ALP—alkaline phosphatase; BSP—bone sialoprotein; IFA—immunofluorescence assay; OCN—osteocalcin; CT—computed tomography; 3D—three-dimensional; CCK-8—Cell Counting Kit-8.

Type of CaP	Biological Samples	Methodological Approach	Main Effects	References
Collagen/HA, HA, biphasic calcium phosphate	Rat MSCs	Cell proliferation (MTT) qRT-PCR	Rapid increase of osteogenic marker gene expression; increased expression of ALP	[154]
Sr-doped CaP	Human MSCs	Cell proliferation (LDH) ALP activity qRT-PCR of BSP II	Increased proliferation; enhanced ALP activity; increased expression of BSP II	[155]
Collagen–nano-HA scaffolds functionalized with microRNA (miRNA)	Human MSCs	qRT-PCR Mineral deposits quantification Calcium deposition IFA	Increased osteogenic markers; mineral deposition	[156]
Ag-doped hydroxyapatite/calcium silicate coating nano-Ti substrates	Mouse preosteobasts (MC3T3-E1 cells)	Cell proliferation (MTT) ALP activity ELISA	Enhanced proliferation; enhanced ALP activity; increased OCN expression	[162]
Co^2+^- and Mg^2+^-doped HA	MG-63 osteoblasts	Flow cytometry IFA	Similar cell-cycle profile as control cells; induced RUNX2 expression	[172]
Biphasic calcium phosphate ceramics	Animal tissue	Histological analysis	Mineralized bone formation	[197]
HA-coated implants	Animal tissue	Removal torque test Histological analysis	Higher removal torque value for HA group; new bone formation with increased density	[198]
HA-coated titanium implants	Animal tissue	Implant stability test	HA-favorable effect on osseointegration	[199]
Ca-doped MgP, HA	Animal tissue	Histological analysis	Bone healing results with complete osseointegration	[204]
Nano-to-submicron hydroxyapatite coatings	MSCs	Cell count and morphology analysis	Reduced cell adhesion	[205]
Sr-doped HA	MC3T3-E1 Animal tissue	Cell proliferation ALP activity Histological analysis	Enhanced proliferation and ALP activity; new bone formation	[213]
Sr-doped HA	Animal tissue	Histological analysis	Higher regeneration efficacy of Sr-doped HA compared to HA and control	[214]
Sr-doped HA	Animal tissue	Micro-CT assessment Histological analysis	Increased bone density around Sr-HA implants; improved trabecular microarchitecture compared to HA	[215,216]
Nanostructured HA scaffolds	Animal tissue	Histological analysis Micro-CT	Superior osteogenic capacity of foamed scaffolds compared to 3D-printed structures	[217]
β-TCP scaffolds	MSCs Animal tissue	Cell proliferation (CCK-8), Micro-CT Histological analysis	Smaller pore sizes; improved bone regeneration	[220]
Nano-HA	Animal tissue	Histological analysis	Bone regeneration similar to commercially available materials	[221]
Nano-HA scaffolds	MSCs Animal tissue	Cell proliferation (MTT) ALP activity qRT-PCR Western blot Micro-CT Histological analysis	Nanostructured HA surfaces promote cell attachment, proliferation, and osteogenic differentiation; enhanced osteo- and angiogenesis in vivo	[227]

**Table 3:** Investigation methods used to assess the angiogenic effects. HUVEC—human umbilical vein endothelial cells; IHC—immunohistochemistry; TCP—tricalcium phosphate; PU—polyurethane; PLGA/PCL—poly(lactic-co-glycolic acid)/poly(ε-caprolactone); HANW—HA nanowire; MS/CS—magnesium silicate core–shell; BMMSC—bone marrow-derived mesenchymal stem cells; CAM—chorioallantoic membrane; IF—immunofluorescence; VEGF—vascular endothelial growth factor; SCPP—Sr-doped calcium polyphosphate; n-ZnO—nano-zinc oxide; HIF-1α—hypoxia-inducible factor.

Type of CaP	Biological Samples	Methodological Approach	Main Effects	References
Mg-doped HA	Co-culture model of HUVECs and MG63	ELISA PCR	Significant effects on bone formation and angiogenesis; Increasing VEGF	[13,238]
	-	IHC	Early angiogenesis followed by early osteogenesis	
Bi-layered scaffold (type I collagen and Mg/HA)	hMSCs	IHC	Stimulating proliferation and differentiation of hMSCs for tissue growth and neo-angiogenesis	[236]
3D scaffold (HA/TCP, PU, PLGA/PCL and collagen I gel.	-	IHC	Stimulating blood vessel formation	[237]
HANW@MS/CS (Magnesium Silicate)	rBMMSCs	SEM RT-qPCR analysis	Mg and Si elements contribute to angiogenic induction, bone formation, and blood vessel formation	[241]
Cu-doped HA	Animal tissue	IHC	CaP scaffold doped with low doses of copper sulfate led to the formation of micro-vessels	[244]
	Animal tissue	SEM	The micro/nano-structure of the Cu5–HA scaffold resulted in more angiogenesis, which formed the new blood vessels	[222]
Sr-doped CaP scaffold	Co-culture model of HUVEC and osteoblasts Animal tissue	Phase-contrast microscopy IHC	Formation of tube-like structure and the expression of platelet endothelial cell adhesion molecule in co-cultured model was better in SCPP scaffold Potential to promote the formation of angiogenesis	[247]
	Animal tissue	IHC	New vessel formation in Matrix-50Sr-HA explants, mainly after 4 weeks of implantations, suggested a positive effect of Sr on angiogenesis	[248]
Co-doped HA	Animal tissue	IHC	Enhanced vascularization in vivo; large blood vessels were predominantly found in Co-doped HA	[249]
Zn-doped HA	-	CAM assay	The number of vessel branches in the modified scaffolds with n-ZnO was significantly higher compared to the modified scaffolds without n-ZnO	[232]
Li-doped HA	BMMSCs (bone-marrow mesenchymal stem cells)	Western blot analysis IHC and IF	HIF-1α and VEGF immunohistochemistry indicated that the hypoxia BMMSCs group had significantly more positive cells than the other three groups	[211]
